# When the limp has a dietary cause: A retrospective study on scurvy in a tertiary Italian pediatric hospital

**DOI:** 10.3389/fped.2022.981908

**Published:** 2022-09-14

**Authors:** Daniela Masci, Chiara Rubino, Massimo Basile, Giuseppe Indolfi, Sandra Trapani

**Affiliations:** ^1^Post-graduate School of Pediatrics, University of Florence, Florence, Italy; ^2^Pediatric Unit, Meyer Children's University Hospital, Florence, Italy; ^3^Radiology Unit, Meyer Children's University Hospital, Florence, Italy; ^4^Department of NEUROFARBA, University of Florence, Florence, Italy; ^5^Department of Health Sciences, Meyer Children's University Hospital, University of Florence, Florence, Italy

**Keywords:** scurvy, vitamin C, children, limping, musculoskeletal pain

## Abstract

The limping child frequently represents a diagnostic challenge. The differential diagnosis is broad and should include vitamin C deficiency. Scurvy, resulting from vitamin C deficiency, is the oldest-known nutritional disorder. Despite its rarity in developed countries, scurvy has been increasingly reported in recent years in pediatric patients, particularly those with autism or neurological disabilities. In the present retrospective study, we describe the clinical, laboratory, and radiological features of 8 patients diagnosed with scurvy in the Pediatrics Unit of Meyer Children's University Hospital, between January 2016 and December 2021. The majority (87%) were males, and the median age was 3.7 years. Half of the patients had comorbidities known to be risk factors for scurvy, while the remaining patients were previously healthy. All the children were admitted for musculoskeletal symptoms, ranging from lower limb pain (87%) to overt limping (87%). Mucocutaneous involvement was observed in 75% cases. Microcytic anemia and elevated inflammatory markers were common laboratory findings. Bone radiographs, performed on all patients, were often interpreted as normal at first, with osteopenia (62%) as the most frequent finding; notably, after re-examination, they were reported as consistent with scurvy in four patients. The most common magnetic resonance imaging findings were multifocal symmetrical increased signal on STIR sequence within metaphysis, with varying degrees of bone marrow enhancement, adjacent periosteal elevation and soft tissue swelling. Differential diagnosis was challenging and frequently required invasive diagnostic procedures like bone marrow biopsy, performed in the first three patients of our series. The median time frame between clinical onset and the final diagnosis was 35 days. Notably, the interval times between admission and diagnosis become progressively shorter during the study period, ranging from 44 to 2 days. Treatment with oral vitamin C led to improvement/resolution of symptoms in all cases. In conclusion, scurvy should be considered in the differential diagnosis in a limping child, performing a detailed dietary history and careful physical examination, looking for mucocutaneous lesions. A quick and correct diagnostic path avoids invasive diagnostic procedures and reduces the risk of long-term complications.

## Introduction

Scurvy, resulting from vitamin C deficiency, is the oldest-known nutritional disorder (1). Nowadays it is considered a rare disease confined to low- or middle-income countries (2). Recently, however, cases of scurvy are re-emerging also in developed countries in wealthy families. Autism spectrum disorders, neurological impairment and malabsorption are considered the main predisposing conditions in children in the modern age (3, 4).

The earliest manifestations of scurvy are non-specific systemic symptoms; however, in the pediatric population, it usually presents with musculoskeletal complaints such as severe lower limb pain, limping and refusal to walk (4–6). Despite this, scurvy is rarely included in the differential diagnosis of a limping child. This often leads to prolonged workup and unnecessary interventions before the right diagnosis is made. In addition, scurvy's clinical features mimic more common diseases such as osteoarticular infectious, malignancy and autoimmune diseases, frequently resulting in misdiagnosis. The diagnosis is based on a combination of clinical and radiographic findings, in a child with a dietary history indicating insufficient intake of vitamin C. A low plasma level of vitamin C is specific for scurvy, though insensitive, as it can be normal if there has been a recent vitamin C consumption in any form. Once started on vitamin C supplementation, patients recover quickly and symptoms usually resolve in 2–4 weeks (5). Resolution of disease manifestations after vitamin C supplementation remains the best evidence of scurvy.

In the present study, we described the clinical characteristics and the diagnostic work up of 8 patients diagnosed with scurvy over the last 6 years in a pediatric tertiary care center. We aimed to highlight the common clinical, laboratory and radiological features of these children, in order to provide useful findings for a correct diagnostic approach.

## Materials and methods

We performed a retrospective study on children diagnosed with scurvy in the Pediatrics Unit of Meyer Children's University Hospital in Florence, Italy, between 1^st^ January 2016 and 31^st^ December 2021. Cases were identified among inpatient hospital records. Patients were included using “scurvy” as discharge diagnosis keyword in Diagnosis Related Groups (DRG) database search. The study describes the authors' experience with eight patients (observational, descriptive research design). Their medical charts were reviewed, in order to collect epidemiological and clinical data, laboratory and radiological features, diagnostic work up, treatment and outcome, as well as time-frame between onset and diagnosis, length of hospitalization and resolution of symptoms. Laboratory investigations including hemoglobin (Hb), Mean Corpuscular Volume (MCV), C–reactive protein (CRP), white cell count, platelet blood count, ferritin, coagulation tests, vitamin D levels on admission were recorded. For each laboratory study, the range, median and interquartile range (IQR) were calculated. Diagnosis of scurvy was based on clinical presentation, radiological findings, vitamin C serum levels lower than 200 μg/dL, and response to treatment. Four cases have been previously reported (7).

## Results

Seven of the eight patients were male, with a median age of 3.7 years (IQR 2.7, range 18 months - 12 years). Four cases (50%) had comorbidities: two patients had autism spectrum disorder (#1 and #6), one patient had cerebral palsy and focal epilepsy (#4), and one had spastic tetraparesis with severe neuro-developmental delay in Micro syndrome and coenzyme q10 deficiency (#8). The remaining four children were previously healthy.

Before being admitted to our hospital, patients had a long duration of symptoms, the median time-frame between clinical onset and hospital admission was 30 days (range 16–241 days), with previous hospitalizations (#2, #3, #8) and access to the emergency department (ED) (range 1–4 accesses to ED before admission).

All patients were admitted for musculoskeletal complaints. Leg pain was the most common presenting symptom (7/8, 87%), associated with low back pain in two patients (2/8, 25%). A limping gait was present in seven out of eight patients (in patient #8 gait was not assessable since she was unable to walk), with complete refusal to bear weight in five patients (5/8, 62%). While three patients reported recent trauma, there was no preceding history of trauma in the remaining five. On admission, three patients presented with fever (#2, #3, and #4). Systemic symptoms, such as fatigue or malaise, were reported in five patients; three had reduced appetite, and almost all patients presented as highly irritable (7/8, 87%). One of the children reported a history of gingival bleeding in the previous weeks, not found on admission.

The clinical examination at admission revealed petechiae and ecchymosis in three patients (#2, #3, and #4), and swollen and bleeding gums in three (#4, #5, and #8). Four patients presented cachectic and pale, with BMI below 5th percentile (#1, #2, #5, #8); in contrast, the nutritional status of the other four was good. Articular examination revealed a swollen limb in four children (#1, #2, #4, and #5), no restriction in range of motion was identified in almost all cases, except for patient #3 who showed restricted hip mobility, keeping a “pithed frog's posture” with semi-flexed knees and hips. Neurological examination revealed reduced patellar tendon reflexes in four patients (#1, #2, #3, and #6), without other abnormal findings, whereas it was normal in the other patients.

During hospitalization, low-grade fever was detected in other two patients (#5 and #8), and three patients developed mucocutaneous involvement with swollen or bleeding gums or petechiae and ecchymosis without evidence of trauma.

Blood tests detected microcytic anemia in seven out of eight patients (median Hb 9.9 g/dL, IQR 1.95, range: 6.9–11.8; median MCV 71.9 fl, IQR 2.42, range: 66.8–74), that in one case required blood transfusion (#2); leukocyte and platelet blood count were normal in all patients. Inflammatory markers were increased in all patients. Median CRP was 1.08 mg/dL (negative if lower than 0,5 mg/dL, IQR 0.95, range: 0.51–8.13); median ESR was 68 mm/h (negative if lower than 20 mm/h, IQR 29.5, range 23–109); median ferritin was 60.5 ng/mL (IQR 31, range 42–164). Fibrinogen was increased in five patients (median 478 mg/dL, IQR 59, range 438–598); lactate dehydrogenase was elevated only in patient #3. Coagulation tests were normal in all patients. Other laboratory findings were unremarkable. All patients underwent an extensive infectious and autoimmune work up, with negative results. Antinuclear antibodies (ANA) were found positive (titer 1:160) in patient #3.

Hip ultrasound was performed on six patients and interpreted as normal, except for patient #6 in which effusion was found in the left hip. The ultrasound was performed before admission to the pediatric ED, when the patient was apyretic and in good general condition; therefore, he was first treated as transient synovitis.

Bone radiographs of lower limbs and pelvis, performed in all patients, were often referred as normal at first, except for osteopenia (5/8, 62%) that was the most frequent finding. After reexamination, they were reported as consistent with scurvy in four patients, documenting metaphyseal spurs with concomitant cupping of the metaphyses (Pelkan spurs, 3/8, 37%), lucent metaphyseal bands (Trümmerfeld zone, 4/8, 50%) and dense metaphyseal line (Frankel's line, 4/8, 50%).

Magnetic resonance imaging (MRI) was performed in seven patients (focused on lower limbs in 5, pelvis in 6, whole body in 1). Common MRI findings were multifocal symmetrical increased signal on STIR sequence within metaphyses (6 cases), with varying degrees of bone marrow enhancement (4 cases), soft tissue swelling (4 cases), and adjacent periosteal elevation (3 cases). A negative MRI of the spine ruled out spinal cord involvement in 4 patients.

**Figure 1:**
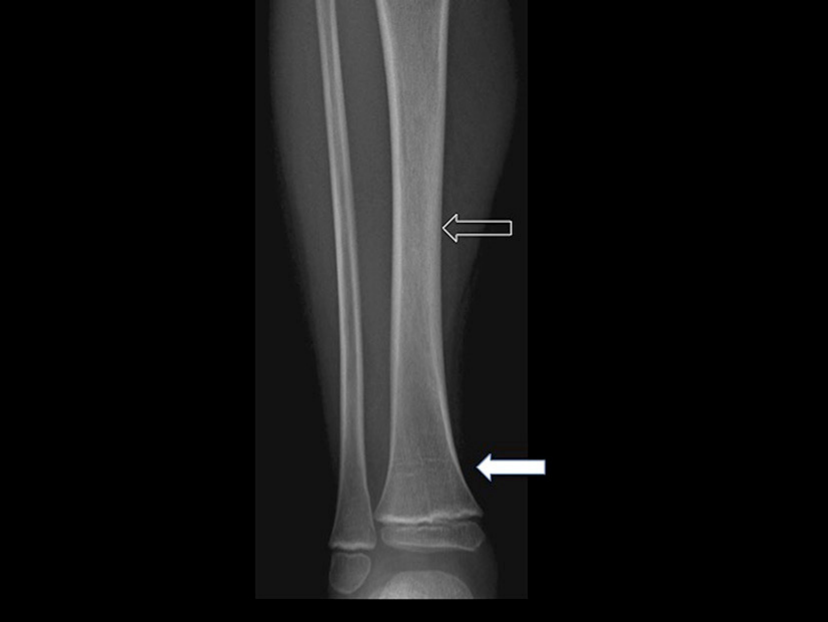
Frontal radiograph of right leg demonstrates multiple transverse growth recovery lines (solid arrow) and inhomogeneous density of tibial diaphysis (open arrow). These findings were not initially appreciated.

**Figure 2:**
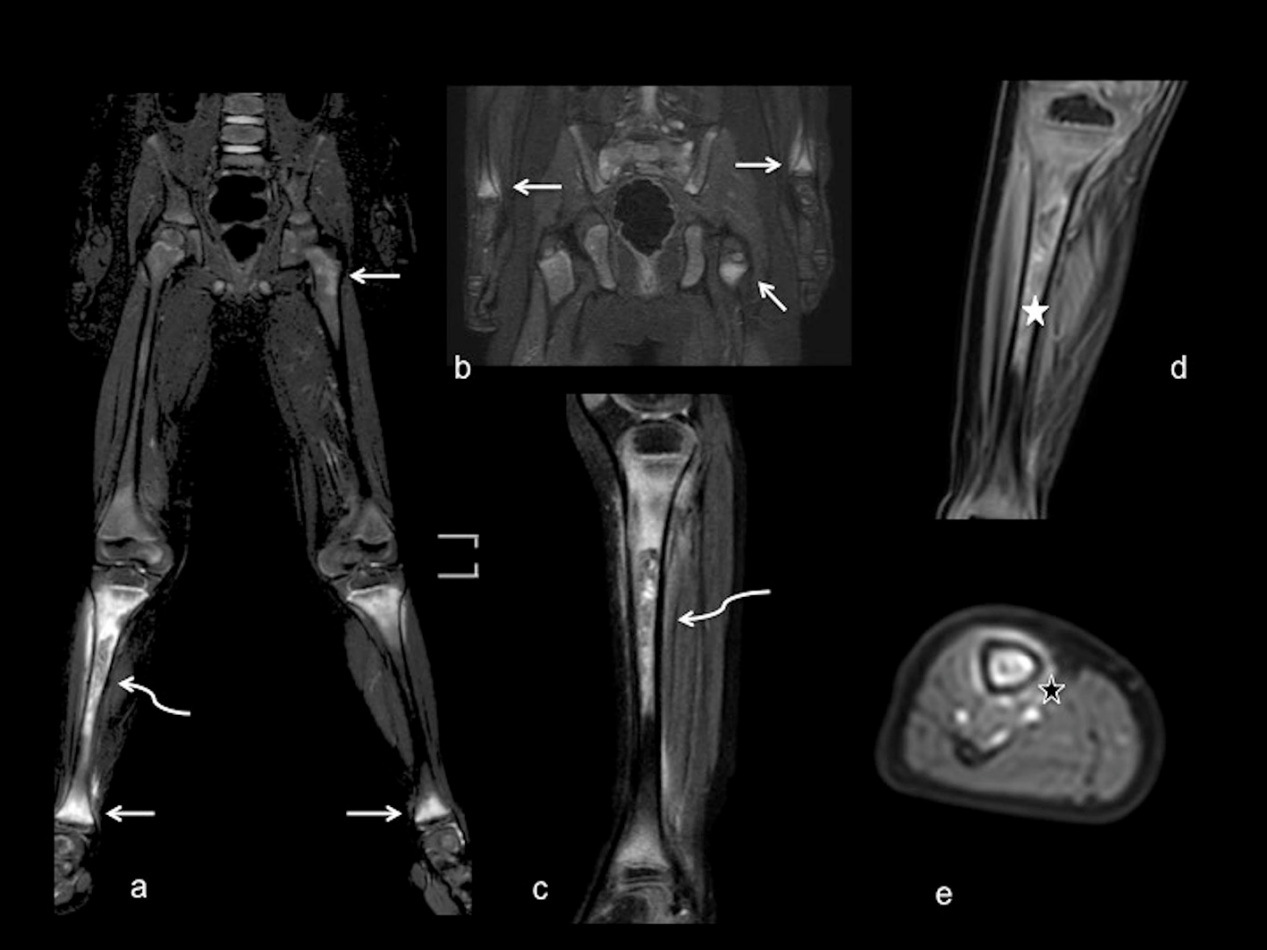
MRI with large field of view. Coronal **(a,b)** and sagittal **(c)** T2-weighted fat saturated views demonstrate abnormal marrow signal of femoral, tibia and wrist metaphyses (white arrows) bilaterally, with more evident involvement of right tibia diaphysis, showing abnormal inhomogeneous signal intensity (curved arrows). Coronal and axial views **(d,e)** after contrast administration show inhomogeneous enhancement of bone marrow (white star) and periosteal tissue (black star), without periosteal collections.

As an example, Figures 1, 2 show radiograph and MRI of patient #5.

In patients #1, #2, #3, and #6, in whom hypoelicitable tendon reflexes were detected, electroneurography was performed and resulted negative in all.

The patients #1 and #2 underwent bone biopsy, which was negative in patient #1, whereas it suggested the diagnosis of myosarcoma in patient #2. Consequently, this patient underwent muscle biopsy, bone marrow aspiration, and a second bone biopsy that ruled out malignancies. Bone marrow aspiration was performed also in patients #1 and #3 and excluded malignancy.

Patient #2 refused to feed, therefore, given his cachectic condition, enteral feeding via nasogastric tube was started. Non-steroidal anti-inflammatory drugs (NSAIDS) were administered to all patients to ease pain, without significant improvement. In 4 cases, the use of opioid analgesics was necessary, with mild improvement of musculoskeletal pain. Empiric intravenous antibiotic therapy was administered to all patients in the initial suspicion of osteoarticular infection (osteomyelitis or septic arthritis).

In all patients an accurate dietary history highlighted restrictive and unbalanced diet, mainly based on carbohydrates. In patients with neurological disabilities (#4 and #8), swallowing difficulties had limited intake to smoothie or homogenized foods; in the year prior to admission both children had excluded or severely limited fruit and vegetable intake. Patients with autism spectrum disorder (#1 and #6) had a history of food aversion of fruits and vegetables started after weaning. In the group without comorbidities, two children started a selective diet after a suspicious reaction to food during weaning; the other two had developed an eating disorder rejecting new foods, in particular fruits and vegetables.

The dietary history, combined with clinical and radiological features, raised the suspicion of scurvy. Thus, vitamin C serum levels were tested and resulted in insufficient concentrations (median 14.50 μg/dL, IQR 38.5, reference range: 460–1,400 μg/dL) in all patients. The median time-frame between admission and vitamin C dosage was six days (IQR 5.5). The interval time between admission and diagnosis become progressively shorter during the study period, ranging from 44 to two days. The average length of hospitalization was found to be 21 days (median 18.5 days, IQR 14.2, range 7–52 days).

Patients were studied for other micronutrient deficiencies: vitamin D was found to be at the lower limits of the normal range in two patients, insufficient in three patients, deficient in three patients. Folate was found to be deficient in all three patients tested. Vitamin A (tested in 3/8), vitamin B12 (tested in 4/8), vitamin E (tested in 2/8) were normal in the small group of patients tested.

Treatment was started with 500 mg of vitamin C daily, administered orally. The supplementation led to improvement of symptoms in all cases, with resolution of cutaneous and mucosal manifestations, improved general conditions and nutritional status, alleviation of pain, and disappearance of limping. Spontaneous bleeding and systemic symptoms (irritability, loss of appetite) recovered quicker, within days, with full recovery in one month in all patients (median three weeks). This was associated with dietary improvement with introduction of new foods in three children. In three patients, laboratory tests were repeated after seven days of treatment, showing CRP and ESR normalization.

The treatment was continued for three months at the same dosage in five patients, the dosage was reduced to 300 mg after the first month in three cases. In addition, in some patients vitamin D (5/8), multivitamin supplementation (3/8), iron supplementation (6/8) were administered orally.

The prompt response to vitamin C supplementation together with the finding of deficient serum vitamin C levels confirmed the clinical suspicion of scurvy.

## Discussion

The limping child frequently represents a diagnostic challenge. The differential diagnosis is broad, ranging from benign and self-limiting to life-threatening diseases. Trauma and infections are the most common causes, but also inflammatory, oncological, neuromuscular, congenital, and hematological pathologies may present with limping. Among unusual etiologies, nutritional deficiencies should also be considered.

Scurvy, resulting from vitamin C deficiency, is the oldest-known nutritional disorder. Nevertheless, due to its rarity in the current era, the diagnosis of scurvy is often forgotten. Pediatric cases of scurvy have been increasingly described in recent years. However, most studies report single or few cases. In the present study, we describe 8 cases diagnosed with scurvy in a tertiary care pediatric hospital in a 6-year period. The above-described cases are representative of the diagnostic challenges and pitfalls of scurvy.

Vitamin C is an essential water-soluble micronutrient, it is involved in several body functions, including collagen biosynthesis, absorption of iron, wound healing and maintenance of blood vessels, cartilage, and bones. Humans are unable to synthesize it, as a result of the loss of an enzyme required in the biosynthetic process, therefore vitamin C must be orally introduced (8, 9).

Insufficient dietary intake may lead to impaired bone formation and vascular fragility, clinically manifesting as scurvy (5).

The prevalence of scurvy in children in developed countries is not known. In the United States, the prevalence of vitamin C deficiency in the general population is about 5.9%, according to the cross-sectional study by the National Health and Nutritional Examination Survey (NHANES) conducted in 2017–2018 on civilians aged more than six years, albeit the majority were clinically asymptomatic. The prevalence of deficiency in the United States is lower in the pediatric population than in adults, though this does not occur in developing countries where low vitamin C deficiency is more frequently observed in children (2, 10–12).

Certain groups of children have a higher risk of ascorbic acid deficiency. The large majority of children with scurvy have underlying conditions predisposing them to nutritional imbalances. Neuropsychiatric or developmental disorders, such as autism spectrum disorders, cerebral palsy and developmental delay, are the most common, as these patients are more prone to restrictive diets owing to sensory issues (3, 13–15). Other diseases may increase the risk of vitamin C deficiency by reducing its absorption (Crohn's, Whipple's, and celiac disease) (16), increasing its requirement (chemotherapy, bone marrow transplant, hemodialysis) (17–19), or accelerating its catabolism (iron overload) (19).

However, scurvy can also occur in healthy children without known risk factors, only due to impaired diet (20–24). Accordingly, half of our patients were previously healthy children with incorrect eating habits. Parental reports of selective eating are frequent in pediatric practice and may be disregarded; picky eating is in fact a common behavior in early childhood that resolves spontaneously for most children (25). However, an overly unbalanced diet can lead to micronutrient deficiencies, as in our cases, so it must be detected and corrected early.

The recommended daily allowance (RDA) of vitamin C is 25 mg for children aged 1–3 years, 30 mg for 4–6 years, 45 mg for 7–10 years, 55–75 mg for 11–18 years, according to the Italian Society for Human Nutrition (26). The global recommendations for vitamin C intake vary greatly between various health authorities, reflecting the different criteria used (27).

The best sources of vitamin C are citrus fruits and fresh vegetables, though cooking and food storage can reduce their vitamin C content. Breast milk and baby formulas also contain an adequate amount of ascorbic acid for infants, around 50–90 mg/L. Cow's milk, on the other hand, has significantly less vitamin C. Therefore, the risk of ascorbic acid deficiency is lower during the first year of life, since infant feeding is predominantly based on breast milk or formula (25, 28).

Most of our patients were around 3 and a half years old. The peak prevalence of picky eating occurs at this age, according to Taylor et al. (25).

In our case series, the majority of patients were male. This gender distribution, coherent with other data reported (5), is unexplained. Epidemiological studies have frequently reported higher vitamin C concentrations in females than in males, but no conclusive gender-related difference in the pharmacokinetics of vitamin C was observed (10, 29, 30).

The first clinical manifestations of scurvy usually appear after 1–3 months of inadequate vitamin C intake (4). The earliest manifestations are non-specific systemic symptoms, such as malaise, asthenia, irritability, loss of appetite and low-grade fever. Later, mucocutaneous signs can appear, including hyperkeratosis, the characteristic perifollicular hemorrhages, petechiae, ecchymosis, corkscrew hairs, gingival swelling and bleeding with teeth loosening. Capillaries become fragile and there is a bleeding tendency. In addition to mucocutaneous bleeding, the bleeding tendency may also manifest as hematuria (31). Musculoskeletal manifestations are the most frequent presenting symptoms in children (5, 6, 32, 33), inducing patients to seek medical attention. Symptoms include arthralgia, myalgia, limb or joint swelling, affecting mainly knees and ankles, hemarthrosis, and muscular hematomas; subperiosteal hematoma can occur during the healing phase. Lower extremities are affected more often, even though any joint can be involved. Leg pain can be so severe that children develop a limping gait, with refusal to walk and inability to bear weight. Infants may present with pseudoparalysis, lying in a “pithed frog position” with semi-flexed hips and knees (34). Defective osteoid matrix and enhanced bone reabsorption, caused by vitamin C deficiency, may lead to spontaneous or inadequate healing of fractures (4).

Scurvy's manifestations may appear discontinuously, and the presence of isolated symptoms can be misleading. In addition, patients may have a good nutritional status, which can further contribute to the diagnostic delay (4, 5).

Other rare clinical signs of scurvy have been described, such as proptosis due to orbital hemorrhage (35), cardiac hypertrophy, pulmonary hypertension, and diminished adrenal and bone marrow functions. Scurvy, if untreated, can be lethal, mainly due to the hemorrhagic diathesis and the difficulty in wound healing, with deaths reported from cerebral hemorrhage and hemopericardium (4, 36–38).

Microcytic anemia and elevation of inflammatory markers are common laboratory findings, as seen in our patients in which all but one had microcytic anemia and all had elevated inflammatory markers, especially ESR with slightly altered CRP. Anemia may be secondary to a combination of bleeding and decreased iron absorption, or abnormal folate metabolism (5, 39–41). Indeed, folate levels should be checked in patients with scurvy, regardless of hemoglobin and mean corpuscular volume values. The inflammatory state may be due to the loss of the antioxidant effect provided by ascorbic acid (14). Anemia with low hematocrit and increased fibrinogen, as seen in our patients, can counteract increasing ESR levels.

These laboratory results may be misleading. In a limping child, elevation of inflammatory markers and anemia might suggest more common diagnoses, such as infectious, inflammatory and oncological diseases. However, these findings should not deter pediatricians from including scurvy in their differential diagnosis. Complete nutritional blood tests should be performed and may reveal further multiple vitamin deficiencies, vitamin D and folate deficiency being the most frequent (42, 43).

Scurvy has typical radiographic findings, especially occurring at the distal ends of the long bones, due to alterations in the osteoid matrix caused by vitamin C deficiency. These include the Frankel line, an irregular but thickened white line in the metaphyseal area, representing the zone of well-calcified cartilage, and the Trümmerfeld zone, a rarefied area secondary to poorly formed trabeculae. Other typical findings are Pelkan spur, resulting from a healed metaphyseal pathological fracture, and the Wimberger ring sign, a thin sclerotic rim surrounding a small lucent epiphysis. Osteopenia is the most common radiographic sign detected in patients with scurvy, though it is not specific (4). These typical imaging findings usually appear after 3–6 months of vitamin C-deficiency, thus they may not be present in the earliest stage, and could not be as clear as it seems even to an expert radiologist (5). As a confirmation of this, imaging studies showed typical alterations only in half of our patients and mostly upon the second review.

While the classical radiographic signs of scurvy are well-described in the medical literature, MRI findings are not so well-delineated and specific. Common MRI features are hypointense signals in T1-weighted sequences and hyperintense in T2-weighted and STIR sequences, mainly within metaphyses. The metaphysis is a site of high turnover, therefore, as a result of poor collagen formation needed for bone growth and repair, it may be affected first in patients with vitamin C deficiency (44). These alterations are often multifocal and symmetrical, suggesting a systemic process. Bone marrow enhancement has been described in several cases. These alterations on MRI might be due to the gelatinous transformation of bone marrow, owing to the replacement of normal marrow elements with hyaluronic acid and water (45). Periosteal elevation and soft tissue alterations may be present, secondary to subperiosteal hemorrhage or edema. These findings, though, are non-specific and can overlap with features of other diseases such as hematological malignancies, metastatic disease, and infectious and non-infectious osteomyelitis; therefore, it is important to keep scurvy in the differential to avoid invasive diagnostic procedures. MRI alterations may be noticeable earlier than radiographic changes and, in the appropriate clinical scenario, may point toward the diagnosis of nutritional deficiency (44–46).

The differential diagnosis in a limping child with these clinical, laboratory and radiological features may be challenging, making the diagnostic pathway full of pitfalls.

According to the history of lower limbs pain and limping, associated in some patients with swollen limb, fever, and elevated inflammatory markers, an osteoarticular infection was initially suspected in all of our patients, thus empiric intravenous antibiotic therapy was administered. Metaphyseal signal abnormalities on MRI may be misinterpreted. Two patients underwent a bone biopsy, but biopsies and blood cultures were negative; however, it is not uncommon for infectious osteomyelitis to show no growth of organisms (47).

Our patients, against this diagnosis, presented low-grade fever, no alterations in white blood count, a slight alteration of CRP with no response to antibiotic therapy. The mucocutaneous signs and the dietary history suggested a diagnosis other than an osteoarticular infection. When performed on an extended field, MRI showed multifocal and symmetrical signal alterations, indicating a systemic process rather than a focal lesion as acute osteomyelitis (45).

The association of symptoms such as limb pain, also during night-time, cachectic appearance and pallor, petechiae, and ecchymosis, gingival hypertrophy, with diffuse bone marrow signal abnormalities on MRI, raised the suspicion of oncologic diseases (48, 49). Therefore, a peripheral blood smear and a bone marrow aspiration were performed in the first three patients to exclude malignancy. Leukemia was also suspected in patient #7. In that case the dietary history collected from the beginning raised the suspicion of nutritional deficiency earlier, therefore bone marrow aspiration was postponed after vitamin C dosage. In the meantime, the patient underwent chest radiography and abdomen ultrasound, which were negative for organomegaly, lymphadenopathy or masses, and a peripheral blood smear showed no blast cells.

As a result of the finding of hypoelicitable reflexes in patients #1, #2, #3 and #6, with gait abnormalities, a neuropathy or a spinal cord disorder were suspected. Electroneurography was normal in all four patients and spine MRI ruled out spinal cord involvement.

Juvenile idiopathic arthritis (JIA) was suspected in patient #3, considering the long history of pain with restricted mobility of the hips, laboratory findings with microcytic anemia, elevated inflammatory markers and ANA positivity. He was treated with NSAIDS (naproxen), without any improvement.

MRI findings of multifocal and symmetric bone marrow abnormalities with periosteal reaction were interpreted as consistent with chronic nonbacterial osteomyelitis (CNO) in patients #1 and #5. This hypothesis was also supported by clinical findings of bone pain and limping associated with elevation of ESR without significantly raised CRP on blood tests (50). A treatment with naproxen was started, without significant improvement. The bone marrow abnormalities and the metaphyseal predilection seen on MRI in scurvy MAY overlap with the imaging findings of CNO (51).

Bone biopsy, performed in patient #1, did not show any bacterial growth, but nor inflammatory changes that should be present in CNO. It is important to consider scurvy in the differential diagnosis to avoid an invasive procedure such as bone biopsy (50). In patient #5, the history of selective and unbalanced diet together with gingival swelling at the physical examination raised the suspicion of scurvy, confirmed by insufficient serum vitamin C levels.

Determination of serum vitamin C levels is the diagnostic gold standard for scurvy: levels lower than 200 μg/dL are considered insufficient and confirm the diagnosis. The test is specific but insensitive, as it may be normal in case of recent ascorbic acid intake in any form. Other ways to estimate the vitamin C body stores are measuring the acid ascorbic level in the buffy-coat of the leukocytes (deficient if lower than 10 mg/10^8^ WBCs), or measuring the urinary excretion of ascorbic acid after parenteral vitamin C infusion. These methods, though, are not readily available. The best evidence of scurvy, in presence of a consistent clinical scenario, remains the resolution of the disease manifestations after vitamin C supplementation (4, 52, 53).

The dosage and length of treatment should be individualized, as there are no standard regimens. Children are usually treated with 100–500 mg of vitamin C daily, for 1 month or until full recovery. Spontaneous bleeding and systemic symptoms usually recover within a few days, with full resolution of clinical manifestations in several weeks (4, 54).

Dietetic regimens should be re-assessed following the acute phase. Indeed, pediatricians and dieticians should verify that the child and the family modify their nutritional intakes. Otherwise, normal vitamin levels are at risk to restore back to insufficient levels after the period of supplementation.

As in other reports (55, 56), our patients had a significant diagnostic delay, with a time-frame between onset and final diagnosis of about 2 months. Notably, the interval times became progressively shorter during the study period, showing that if we consider scurvy among the causes of limping, a final diagnosis can be made in a few days.

## Conclusion

In pediatric scurvy, the presenting complaints are typically musculoskeletal symptoms. Scurvy must be considered in the differential diagnosis of a child with a limp, not only in children with known risk factors but also in healthy ones. During the assessment of a limping child, an accurate dietary history is fundamental in order to detect potential nutritional deficiency, together with a careful physical examination looking for typical mucocutaneous signs of scurvy such as hyperkeratosis, perifollicular hemorrhages, petechiae, ecchymosis, corkscrew hairs, gingival swelling and bleeding. Microcytic anemia and ESR elevation with only slight alteration of CRP are common laboratory findings. Imaging studies, if performed by expert radiologists, may reveal the typical features of scurvy. Common MRI findings are multifocal symmetrical increased signal on STIR sequence within metaphyses, with varying degrees of bone marrow enhancement, with adjacent periosteal elevation and soft tissue swelling. Although not as specific as radiographic changes, MRI alterations may be noticeable earlier. Differential diagnosis can be challenging because scurvy can mimic common and severe infectious, oncologic, inflammatory conditions. This often leads to long hospitalization and unnecessary procedures. Scurvy's manifestations are reversible with vitamin C supplementation, so any delay in diagnosis contributes to unnecessary pain and clinical morbidity. A correct diagnostic path avoids invasive diagnostic procedures and reduces the risk of long-term complications.

## Data availability statement

The raw data supporting the conclusions of this article will be made available by the authors, without undue reservation.

## Ethics statement

Ethical review and approval was not required for the study on human participants in accordance with the local legislation and institutional requirements. Written informed consent to participate in this study was provided by the participants' legal guardian/next of kin.

## Author contributions

ST conceived the study. DM collected data, conducted literature research, and wrote the first draft. CR, ST, MB, and GI critically revised the draft. All authors contributed to the article and approved the submitted version.

## Conflict of interest

The authors declare that the research was conducted in the absence of any commercial or financial relationships that could be construed as a potential conflict of interest.

## Publisher's note

All claims expressed in this article are solely those of the authors and do not necessarily represent those of their affiliated organizations, or those of the publisher, the editors and the reviewers. Any product that may be evaluated in this article, or claim that may be made by its manufacturer, is not guaranteed or endorsed by the publisher.
